# Financial Exploitation Among Older Adults in Israel: Exploring the Role of Culture

**DOI:** 10.1093/geroni/igaf122.104

**Published:** 2025-12-31

**Authors:** Gali Weissberger, Yoav Bergman

**Affiliations:** Bar-Ilan University, Ramat Gan, HaMerkaz, Israel; Ashkelon Academic College, Ashkelon, HaDarom, Israel

## Abstract

Financial exploitation (FE) of older adults results in devastating social and psychological consequences. However, few studies have considered culture as a contextual factor that may modify risk factors and consequences of FE. In this study, we examined the psychosocial correlates of experienced FE among two main cultural groups in Israel: the majority group of Jewish Israelis, and the minority group of Arab Israelis. Through convenience sampling techniques, 357 Israeli older adults (M age = 67.89, SD = 6.21, 72.3% female; 13.4% Arab) were recruited into the study. Participants self-reported whether or not they experienced FE after the age of 50, responded to demographic questions, and completed psychosocial questionnaires (loneliness, social support, and psychological distress). A MANCOVA model tested whether the association between FE and psychosocial factors varies by cultural group. There were 79 participants (22% of the sample) who self-reported an experience of FE. Results demonstrated a main effect of FE on the combined dependent variables (F(3, 346) =10.98, p < 0.001); there was no main effect of cultural group. A FE by cultural group interaction was found (F(3, 346) =3.78, p = 0.011). Follow-up ANCOVA models revealed that the negative effect of FE on psychosocial functioning, and specifically loneliness and psychological distress, was stronger for Israeli Arabs compared to Israeli Jews. These findings suggest that Israeli Arabs who experience FE may be in particular need of support following a FE experience, and highlight the importance of considering culture as a contextual factor that may modify risk factors and consequences of FE.

